# Zoledronic acid increases docetaxel cytotoxicity through pMEK and Mcl-1 inhibition in a hormone-sensitive prostate carcinoma cell line

**DOI:** 10.1186/1479-5876-6-43

**Published:** 2008-08-08

**Authors:** Francesco Fabbri, Giovanni Brigliadori, Silvia Carloni, Paola Ulivi, Ivan Vannini, Anna Tesei, Rosella Silvestrini, Dino Amadori, Wainer Zoli

**Affiliations:** 1 Biosciences Laboratory, Istituto Scientifico Romagnolo per lo Studio e la Cura dei Tumori (I.R.S.T.), Meldola, Italy; 2 Department of Medical Oncology, Istituto Scientifico Romagnolo per lo Studio e la Cura dei Tumori (I.R.S.T.), Meldola, Italy

## Abstract

**Background:**

In prostate cancer, the identification of drug combinations that could reduce the tumor cell population and rapidly eradicate hormone-resistant cells potentially present would be a remarkable breakthrough in the treatment of this disease.

**Methods:**

The study was performed on a hormone-sensitive prostate cancer cell line (LNCaP) grown in normal or hormone-deprived charcoal-stripped (c.s.) medium. Cell viability and apoptosis were assessed by SRB assay and Annexin-V/TUNEL assays, respectively. Activated caspase-3, p21, pMEK and MCL-1 expression levels were detected by western blotting.

**Results:**

The simultaneous exposure of zoledronic acid [100 μM] and docetaxel [0.01 μM] for 1 h followed by treatment with zoledronic acid for 72, 96 or 120 h produced a high synergistic interaction (R index = 5.1) with a strong decrease in cell viability. This cytotoxic effect was associated with a high induction of apoptosis in both LNCaP and in c.s. LNCaP cells. The induction of apoptosis was paralleled by a decrease in pMEK and Mcl-1 expression.

**Conclusion:**

The zoledronic acid-docetaxel combination produced a highly significant synergistic effect on the LNCaP cell line grown in normal or hormone-deprived medium, the principal molecular mechanisms involved being apoptosis and decreased pMEK and Mcl-1 expression. This experimentally derived schedule would seem to prevent the selection and amplification of hormone-resistant cell clones and could thus be potentially used alongside standard androgen deprivation therapy in the management of hormone-sensitive prostate carcinoma.

## Introduction

The overall incidence of prostate cancer, one of the most common lethal malignancies and the second cause of cancer mortality in males, is gradually increasing in western countries. In the early stages of the disease, surgery, radiotherapy and/or androgen deprivation are the most effective clinical therapies. In particular, hormonal therapy leads to remission which typically lasts from 2 to 3 years. However, prostate cancer frequently metastasizes to bone and almost invariably progresses to an androgen-independent state, with a poor prognosis and a median survival that varies from 10 to 20 months [1]. Notwithstanding the introduction of new chemotherapeutic agents, the life expectancy of patients with advanced prostate cancer is still limited. The development of new drugs or the identification of novel drug combinations which could reduce the development of endocrine-refractory cell clones thus remain important goals.

It has been shown that docetaxel (Doc) exerts a potent cytotoxic effect *in vitro *and considerably prolongs survival in patients with advanced prostate cancer [2,3]. At the same time, zoledronic acid (Zol) has proven to be capable of preventing tumor growth in different *in vitro *models [4] and has shown significant clinical potential for reducing cancer-related bone lesions and inhibiting bone re-absorption [5].

The Ras/Raf/MEK/ERK signalling cascade is one of the most important intracellular pathways controlling cell proliferation, differentiation and cell death, and appears to be involved in prostate cancer drug resistance [6,7]. Moreover, in different experimental models, it has been shown that inhibition of at least one of these regulatory proteins may induce apoptosis through the downregulation of the anti-apoptotic protein Mcl-1, a member of the Bcl-2 family [8,9]. Mcl-1 is expressed in a fairly high percentage of prostate tumors [10-12], and the inhibition of Ras/Raf/MEK/ERK-mediated signals, and consequently of Mcl-1 expression, could therefore also be a key objective in the treatment of hormone-sensitive prostate cancer cells, as shown by Cavarretta et al [13].

The aim of the present study was to examine the *in vitro *activity of Zol and low Doc concentrations, alone or in combination, and to explore the molecular mechanisms underlying treatment-related cell proliferation and apoptosis, especially in relation to MEK and Mcl-1 expression. Very low Doc concentrations were chosen so as not to preclude the use of the taxane at conventional doses as second-line treatment in more advanced disease. Moreover, in order to approximate clinical conditions, the study was performed on cells grown in normal or hormone-deprived medium and on the same cell line after pretreatment with the taxane.

## Materials and methods

### Cell culture

The studies were performed on a hormone-sensitive prostate cancer cell line, LNCaP, obtained from the American Type Culture Collection (Rockville, MD). The cell line was maintained as a monolayer at 37°C and subcultured weekly. Culture medium was composed of RPMI 1640 supplemented with 10% fetal calf serum and 1% glutamine (Mascia Brunelli s.p.a., Milan, Italy). Cells were used in the exponential growth phase in all the experiments. Depending on the experimental setting, LNCaP cells were seeded in RPMI 1640 medium containing either 10% fetal calf serum or charcoal-stripped 10% fetal calf serum. Doc pre-treated LNCaP cells were generated by exposing the cell line to 0.001 μM of Doc for 1 h once a week for one month, which produced cells resistant to this dose of taxane. Thereafter, these cells were maintained in culture medium containing 0.001 μM of the taxane.

### Drugs

Docetaxel (Taxotere^®^), kindly supplied by Aventis Pharma, was solubilized and stored at a concentration of 12.6 mM in 13% ethanol at 4°C and diluted in medium before use. The final concentration of ethanol never exceeded 0.01% and therefore had no effect on cell growth or viability. Control cells were exposed to the same amount of solvent. Zoledronic acid (Zometa^®^) (Zol), kindly provided by Novartis, was solubilized and stored at a concentration of 25 mM in sterile water at -20°C and diluted in medium before use.

### Chemosensitivity assay

Sulforhodamine B (SRB) assay was used according to the method by Skehan et al. [14]. Briefly, cells were collected by trypsinization, counted and plated at a density of 5,000 cells/well in 96-well flat-bottomed microtiter plates (100 μl of cell suspension/well). In the chemosensitivity assay, experiments were run in octuplicate, and each experiment was repeated three times. The optical density (OD) of cells was determined at a wavelength of 540 nm by a colorimetric plate reader. Growth inhibition and cytocidal effect of drugs were calculated according to the formula reported by Monks et al [15]: [(OD_treated _- OD_zero_)/(OD_control _- OD_zero_)] × 100%, when OD_treated _is > to OD_zero_. If OD_treated _is above OD_zero_, treatment has induced a cytostatic effect, whereas if OD_treated _is below OD_zero_, cell killing has occurred. The OD_zero _depicts the cell number at the moment of drug addition, the OD_control _reflects the cell number in untreated wells and the OD_treated _reflects the cell number in treated wells on the day of the assay.

### Single drug exposure

Cells were exposed for 1 h to 0.001-, 0.01-, 0.1- or 1.0-μM concentrations of Doc followed by a 72-, 96- or 120-h culture in drug-free medium. Zol treatment consisted of continuous exposure of 50-, 100-, or 200-μM concentrations for 72, 96 or 120 h.

### Drug combinations

The following treatment schedules were utilized (Table 1):

**Table 1:** Treatment schedules in LNCaP hormone-sensitive cell line

Doc* + Zol (100 μM) 1 h → Zol (100 μM) 72, 96, 120 h
Zol (100 μM) 72, 96, 120 h → Doc* + Zol (100 μM) 1 h

* 0.001, 0.01, or 0.1 μM

1. Simultaneous exposure to Zol 100 μM and Doc 0.001, 0.01 or 0.1 μM for 1 h followed by Zol exposure for 72, 96 or 120 h;

2. Continuous exposure to Zol for 72, 96 or 120 h followed by simultaneous exposure to Zol 100 μM and Doc 0.001, 0.01 or 0.1 μM for 1 h.

Cytotoxic activity was evaluated immediately after the end of drug exposure.

### Drug interaction analysis

Kern et al.'s method [16], subsequently modified by Romanelli et al. [17], was used to evaluate the interaction between drugs. In brief, the expected cell survival (*S*_exp_, defined as the product of the survival observed with drug A alone and the survival observed with drug B alone) and the observed cell survival (*S*_obs_) for the combination of A and B were used to construct an R index (RI): RI = *S*_exp_/*S*_obs_. An RI of ≤ 0.5 indicated the absence of synergism or antagonism. Synergism was defined as any value of RI > 1.5. In all experiments, the standard deviation did not exceed 10%. Therefore, only differences of ≥ 0.5 from unity in RI values were considered significant.

### Flow cytometry

After different drug exposures, medium was removed and cells were detached from the flasks by trypsin treatment, washed twice with PBS and stained according to the different methods specified below. Flow cytometric analysis was performed using a FACS Canto flow cytometer (Becton Dickinson, San Diego, CA). Data acquisition and analysis were performed using FACSDiva software (Becton Dickinson). Samples were run in triplicate and 10,000 events were collected for each replica. Data were the average of three experiments, with errors under 5%.

### Apoptosis

#### TUNEL assay

Cells were fixed in 1% paraformaldehyde in PBS on ice for 15 min, suspended in ice cold ethanol (70%) and stored overnight at -20°C. Cells were then washed twice in PBS and resuspended in PBS containing 0.1% Triton X-100 for 5 min at 4°C. Thereafter, samples were incubated in 50 μl of solution containing TdT and FITC-conjugated dUTP deoxynucleotides 1:1 (Roche Diagnostic GmbH, Mannheim, Germany) in a humidified atmosphere for 90 min at 37°C in the dark, washed in PBS, counterstained with propidium iodide (2.5 μg/ml, MP Biomedicals, Verona, Italy) and RNAse (10 Kunits/ml, Sigma Aldrich, Milan, Italy) for 30 min at 4°C in the dark and analyzed by flow cytometry.

#### Annexin-V assay

Cells were harvested, washed once in PBS and incubated with 10 μl/ml Annexin V-FITC in binding buffer (Bender MedSystems, Vienna, Austria) for 15 min at 37°C in a humidified atmosphere in the dark. Cells were then washed in PBS and suspended in binding buffer. Immediately before flow cytometric analysis, propidium iodide was added to a final concentration of 5 μg/ml to distinguish between total apoptotic cells (Ann-V + and PI - or +) and necrotic cells (Ann-V - and PI +). For each sample, 15,000 events were recorded.

### Western blot

Cells were lysed and proteins were denaturated, separated on 10% SDS-polyacrylamide gel and electroblotted onto Hybond-C extra membrane (Amersham Pharmacia Biotech, Cologna Monzese, Italy). The membrane was stained with Ponceau S (Sigma Aldrich) to verify equal amounts of sample loading and then incubated for 2 h at room temperature with T-PBS 5% non fat dry milk. The membrane was probed overnight at 4°C with the antibody, after which horseradish peroxidise-conjugated secondary antibody diluted 1:1,000 (Dako Corporation, Glostrup, Denmark) was added. Bound antibodies were detected by enhanced chemiluminescence (ECL) using an ECL kit (Amersham Pharmacia Biotech). The following primary antibodies were used: p21 (monoclonal antibody, dilution 1:250) (BioOptica, Milan, Italy), caspase-3 (polyclonal antibody, dilution 1:500) (Cell Signalling Technology, Inc., Beverly, MA), pMEK (monoclonal antibody, dilution 1:1000) (Cell Signalling Technology), actin (polyclonal antibody, dilution 1:5000) (Sigma Aldrich) and Mcl-1 (monoclonal antibody, dilution 1:100) (BD, Pharmingen, San Diego, CA). Quantitative analysis was carried out with Quantiscan software (Biosoft, Cambridge, UK).

### Morphological investigation

After different drug exposures, medium was removed, cells were detached from the flasks by trypsin treatment, washed twice with PBS, fixed in ethanol (70%), stained with the cell-permeable dyes 4',6-DAPI (Molecular Probes, Leiden, The Netherlands) and SRB (Sigma Aldrich) and then examined by fluorescence photomicroscope (Zeiss, Axioscope 40) to visualize chromatin condensation and/or fragmentation typical of apoptotic cells.

### Statistical analysis

Differences between treatments, in terms of dose response and apoptosis, were determined using the Student's t test for unpaired observations. p < 0.05 was considered significant.

## Results

### Single drug activity

**Figure 1:**
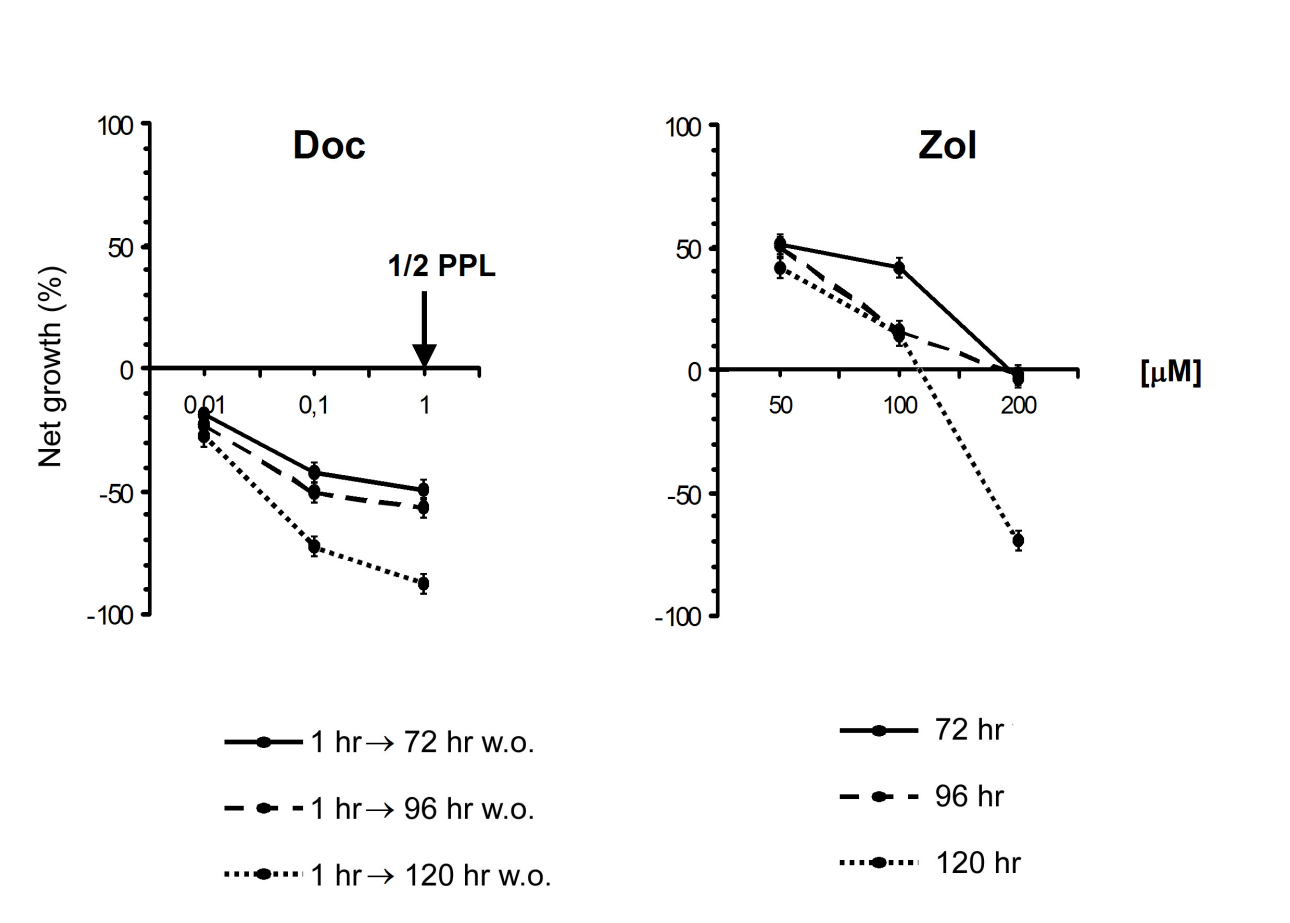
**Dose effect curves of Doc and Zol in LNCaP hormone-sensitive cell line**. Net cell growth was measured by SRB assay at the end of different washout (w.o.) times after a 1-h exposure to scalar concentrations of Doc (0.01, 0.1, 1.0 μM – left diagram) and after continuous exposure to scalar concentrations of Zol (50, 100, 200 μM – right diagram). Error bars represent the mean standard deviation.

Cytotoxicity was assessed at scalar drug concentrations and after different exposure times. Doc produced a strong cytotoxic effect after all treatment schedules and the 50% lethal concentration (LC_50_) was reached 120 h after a 1-h exposure to a 0.057-μM concentration. Although Zol treatment also produced a cytotoxic effect, LC_50 _was only reached after a 120-h exposure to a concentration of 176.70 μM (Figure 1).

### Drug combinations

**Figure 2:**
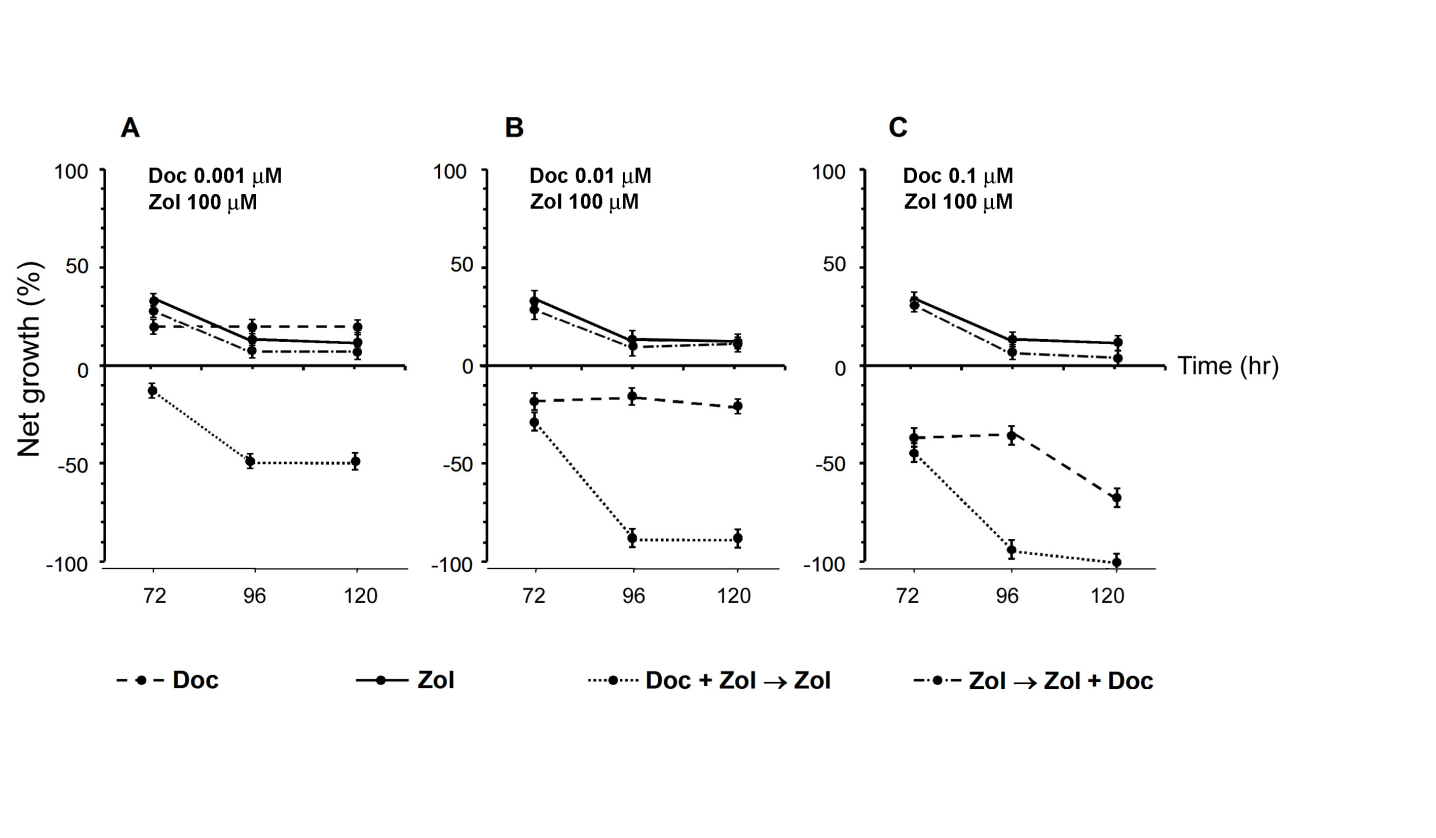
**Comparison between different drug schedules**. Dashed line: activity observed 72, 96 and 120 h after a 1-h exposure to low Doc concentrations (0.001, 0.01 and 0.1 μM in diagrams A, B and C, respectively). Solid line: activity detected after a 72-, 96- and 120-h continuous exposure to Zol 100 μM. Dotted line: activity observed after a 1-h concomitant exposure to Zol 100 μM and low Doc concentrations (0.001, 0.01 and 0.1 μM, in diagrams A, B and C, respectively) followed by prolonged exposure to Zol 100 μM. Dash-dotted line: activity observed after prolonged exposure to Zol 100 μM followed by a 1-h concomitant exposure to Zol 100 μM and low Doc concentrations (0.001, 0.01 and 0.1 μM, in diagrams A, B and C, respectively). Error bars represent the mean standard deviation.

After preliminary experiments, the efficacy of two different drug combination schedules was further explored (Table 1). Simultaneous exposure to Zol (100 μM) and Doc (0.001 μM) for 1 h followed by Zol exposure for 72, 96 or 120 h produced a strong cytocidal effect, and the LC_50 _was reached after 96 h of continuous exposure (Figure 2A). At the higher Doc concentrations (0.01 and 0.1 μM), cytocidal activity further increased, exceeding the LC_90 _(Figure 2B–C). Starting from a 0.01-μM Doc concentration, the combination schedule produced an important synergistic effect which yielded an R index of 5.1. The highest Doc concentration (0.1 μM) did not produce any further significant synergistic interaction**s **and was therefore not tested in subsequent experiments. The schedule consisting of Zol followed by simultaneous exposure to Zol and Doc induced only a very weak additive effect with respect to Zol used alone (Figure 2A–B–C).

### Apoptosis

Assessment of apoptosis by TUNEL assay showed that low-dose Doc induced a small percentage of apoptotic cells which never exceeded 10% at either the longest exposure time (120 h) or the highest drug concentration (0.01 μM). Conversely, Zol 100 μM induced a statistically significant, exposure time-dependent increment in apoptotic cells between 4- and 20-fold higher than that detected in untreated cells (Table 2).

**Table 2:** Apoptotic cells (%) in LNCaP line by TUNEL assay

		Observation time		
		72 h	96 h	120 h
**Control**		1.5	2.0	2.0
**Doc 1 h**	0.001 μM	1.3	1.1	4.8
	0.01 μM	2.5	3.8	9.2
**Zol continuous** ** exposure**	100 μM	4.9	11.5	37.8*
**Doc **(0.001 μM) + **Zol **(100 **μ**M) 1 h → **Zol **(100 μM)		11.2	20.1	30.8*
**Doc **(0.01 μM) + **Zol **(100 **μ**M) 1 h → **Zol **(100 μM)		40.2	41.6	66.5*

*Significance at p < 0.05 by t test

**Figure 3:**
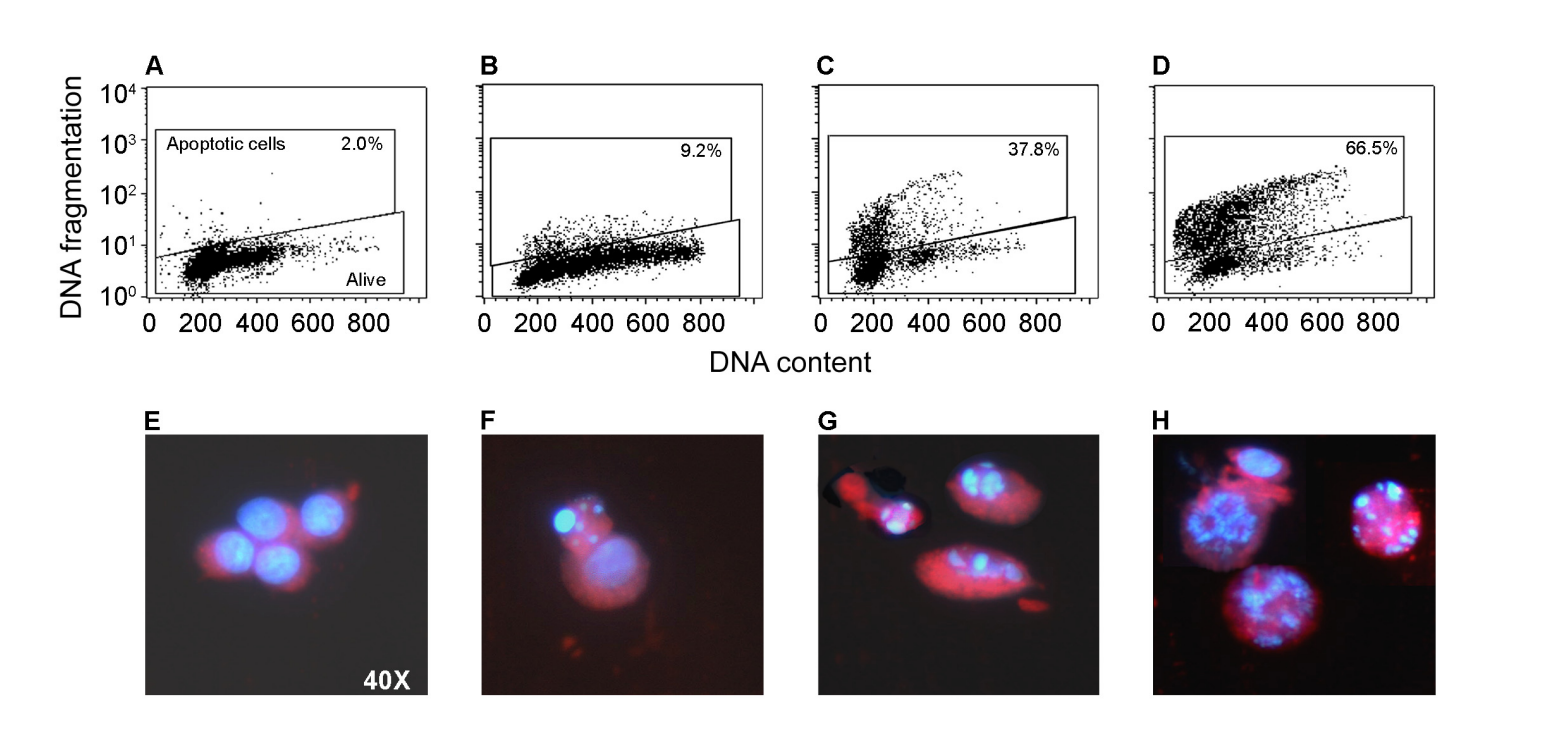
**Representative cytofluorimetric dot plots of one of the TUNEL experiments and characteristic morphologic images of apoptotic cells observed after drug exposure.** A and E, untreated cells; B and F, after a 1-h exposure to Doc 0.01 μM followed by a 120-h w.o.; C and G, after a 120-h continuous exposure to Zol 100 μM; D and H, after exposure to the combination Doc + Zol → Zol.

**Figure 4:**
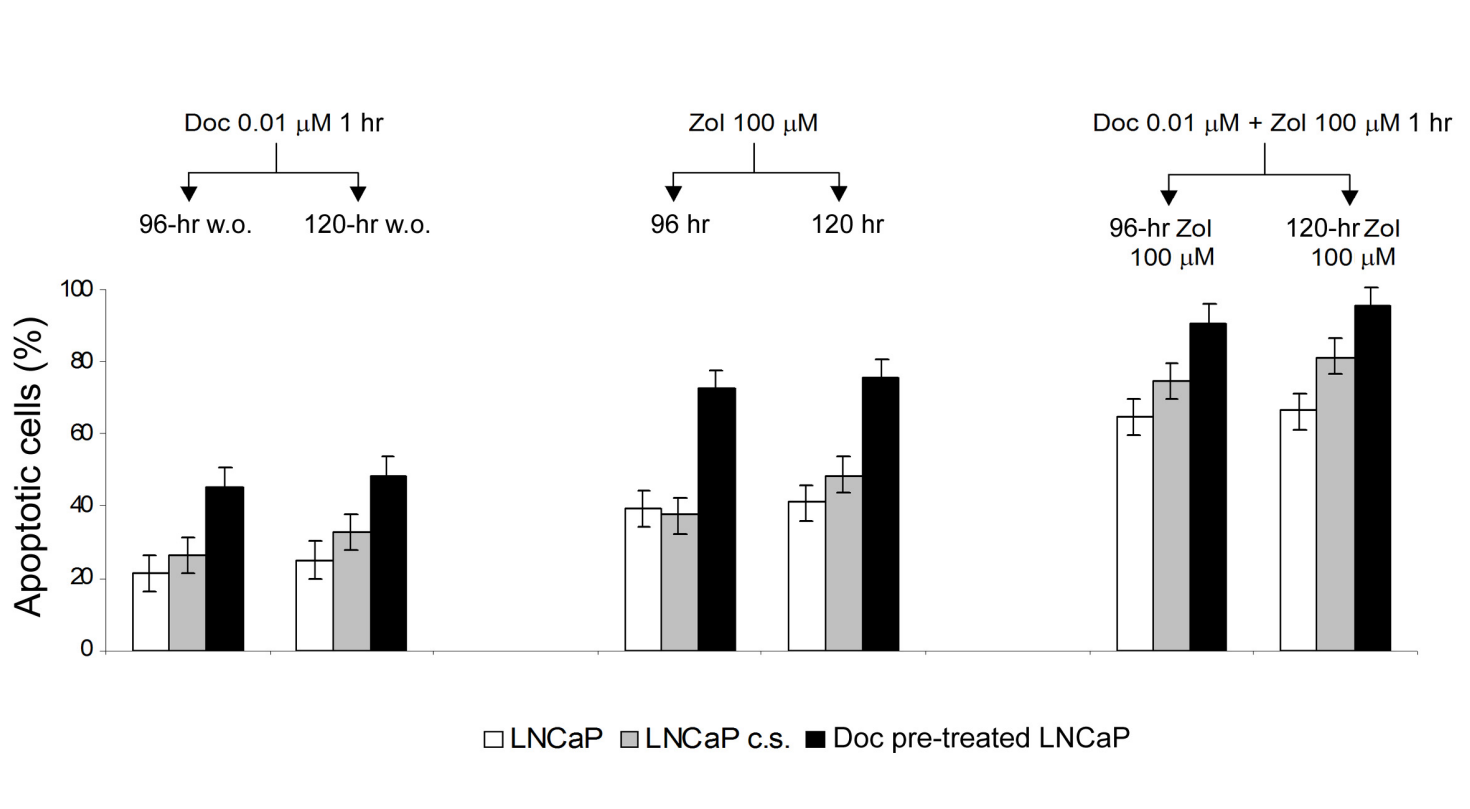
**Apoptotic cells (%) measured by Ann-V assay in LNCaP line grown in normal medium (white bars), charcoal-stripped (c.s.) hormone-deprived medium (grey bars) and normal medium after taxane pre-treatment (black bars)**. Cells were analyzed after a 1-h exposure to Doc 0.01 μM followed by a 96- or 120-h w.o. (left histogram bars), after a 96- or 120-h continuous exposure to Zol 100 μM (center histogram bars), and after exposure to the Doc-Zol combination (right histogram bars). The percentage of apoptosis in untreated cells never exceeded 10%. Error bars represent the mean standard deviation.

The schedule using Doc 0.001 μM did not produce a significantly higher number of apoptotic cells with respect to treatment with Zol alone. Conversely, the drug combination using a 0.01-μM Doc concentration induced a significant increase in the percentage of TUNEL-positive cells ranging from 40% at 72 h to 66% at 120 h (Table 2). Similar results for the TUNEL assay were observed regardless of the type of medium used and were confirmed by morphological evaluation of cells exposed to the aforementioned treatments (Figure 3). Apoptosis evaluation by ANN-V assay in the LNCaP line grown in different hormone culture conditions showed that the number of apoptotic cells progressively increased after Doc, Zol or combined drug exposure from about 20 to 70% at any of the times considered, and once again this increase was similar regardless of the type of medium used (Figure 4). The number of apoptotic cells also showed a similar trend in taxane pre-treated LNCaP cells, but reached a significantly higher level (90%) after the drug sequence exposures.

### Interference in apoptosis- and cell proliferation-related markers

**Figure 5:**
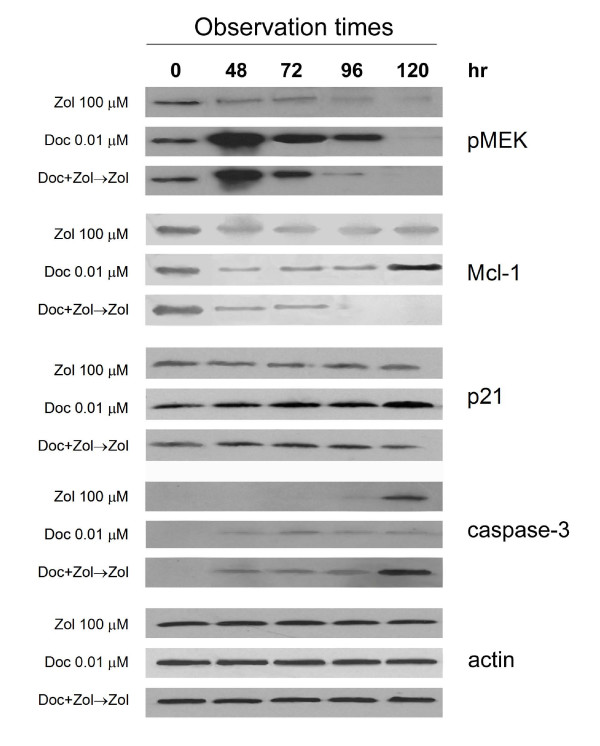
**pMEK, Mcl-1, p21 and active caspase-3 protein expression observed by western blot after exposure to different drug schedules**. In the combination schedule, drug concentrations were the same as those used for single drug exposures.

The expression of the pMEK protein kinase was progressively downregulated from 2- to 10-fold by the bisphosphonate and completely disappeared after 96 h of continuous exposure to the drug (Figure 5). Conversely, a 1-h treatment with Doc induced a 7-fold increase in pMEK expression after 48 h, which then progressively decreased and disappeared at 120 h. After the synergic drug sequence exposure, a 4-fold increase in pMEK expression with respect to the control was also observed, which, however, completely disappeared after 96 h.

The expression of Mcl-1 protein was downregulated up to 3- and 5-fold by Zol and Doc, respectively. However, whilst these inhibiting effects persisted in the presence of the bisphosphonate for up to 120 h, a 1.5-fold upregulation of Mcl-1 was observed after treatment with the taxane with respect to the control, showing a complementary profile to that of pMEK. The drug sequence exposure not only prevented the late upregulation observed after treatment with Doc, but also induced the total disappearance of Mcl-1 protein expression. No important variations in pMEK or Mcl-1 expression with respect to the control were observed after exposure times of < 48 h (data not shown).

Whilst p21 expression was not affected by exposure to Zol, it increased up to 2-fold after Doc treatment. Conversely, p21 expression showed a > 1.5-fold decrease after 120 h of the combination treatment.

The active form of caspase-3 was detected around 120 h after bisphosphonate exposure, whereas it was observed earlier and at lower levels after Doc treatment. Conversely, after the drug sequence exposure, its expression profile was quantitatively and qualitatively the sum of the expression detected after exposure to the single drugs.

All the aforementioned alterations in marker expression were observed regardless of the type of medium used.

## Discussion

In the present study, docetaxel and zoledronic acid induced noteworthy cytostatic and cytocidal effects on the hormone-sensitive prostate cancer cell line, LNCaP, in agreement with results from previous papers [18-21]. In particular, the highest inhibition of cell proliferation was observed after Doc exposure and was already evident at concentrations 200-fold lower than the plasma peak level.

Interestingly, among the different drug schedules tested, a short, concomitant exposure to Zol and low Doc concentrations followed by a prolonged Zol exposure proved to be the most effective combination. In particular, this schedule produced an elevated synergistic interaction and a strong decrease in cell viability. Conversely, the combination of Zol followed by simultaneous exposure to Zol and Doc induced only a weak additive effect, which may be a result of Zol triggering apoptosis directly in the G0/1 phase and leaving virtually no cells in G2/M, which is known to be Doc's main target.

Drug concentrations and exposure times utilized in our study were chosen on the basis of both literature data [19-28] and results from preclinical investigations carried out in our laboratory [29,30] to identify dosages and timing that would have a significant impact on hormone-sensitive prostate cancer cell survival. Our results suggest that this therapeutic approach could be useful as first-line treatment of hormone-sensitive prostate cancer. Furthermore, notwithstanding the obvious differences between our experimental system and a clinical setting of metastatic prostate cancer, the cell model (LNCaP) we used was originally isolated from a metastatic lymph node [31] and can therefore be considered fairly representative of hormone-sensitive metastatic disease. This would seem to suggest the potential clinical applicability of our results for patients with hormone-sensitive metastatic prostate cancer.

The two drugs induced mainly independent effects on apoptosis and cell proliferation. In fact, low Doc concentrations produced a high cytostatic effect, probably linked to cell cycle arrest, but induced a relatively low fraction of apoptotic cells, whereas Zol exerted a predominantly high cytotoxic and pro-apoptotic effect on the cell line. Despite this apparent incongruence, sequential exposure to the two-drug sequence caused a considerable increment in the percentage of apoptotic cells, reaching values between 2- and 6-fold higher than those observed after single drug exposure, and further confirming the synergistic cytotoxic effect.

We also evaluated drug activity in cells grown in hormone-deprived medium, thus approximating the standard clinical setting in which prostate cancer patients are treated with androgen deprivation therapy. Under these conditions, the percentage of apoptotic cells after exposure to various treatment schedules was similar to that observed in cells grown in normal medium, indicating that a hormone-deprived environment should not, in theory, compromise the activity of chemotherapy. Moreover, single drug or combination activity was maintained in LNCaP cells pre-treated with low Doc concentrations. Our results therefore suggest that treatment with low taxane doses would not preclude response to conventional docetaxel doses used in the clinical treatment of advanced, hormone-refractory tumors.

Cell reactions to single drugs and their association were paralleled by alterations in the expression of proteins involved in cell proliferation and apoptosis. Zol treatment generally induced a prolonged downregulation of both pMEK, known to be involved in cell proliferation signal transduction [8], and the anti-apoptotic protein, Mcl-1 [9]. Furthermore, the bisphosphonate did not cause any major changes in p21 expression, known to be related to cell cycle arrest and Doc resistance [32], and triggered the activation of the pro-apoptotic caspase-3 only after a long exposure. Conversely, low Doc concentrations induced a less evident downregulation of pMEK, an upregulation of Mcl-1 at the longest washout times, a clear increase in p21 expression, and a slight increase in the active form of caspase-3. Following the synergistic drug sequence used in our study, pMEK and Mcl-1 expression strongly decreased and p21 expression was slightly downsized, whereas activated caspase-3 showed early and marked upregulation. These results suggest that treatment with low Doc concentrations activates apoptotic processes, which, however, may not be completed due to the concomitant triggering of pMEK-, Mcl-1- and p21-mediated survival pathways. Conversely, Zol seems to induce apoptosis and downregulate Doc-elicited anti-apoptotic mechanisms, bringing to an end the cell death processes initiated by the taxane.

Targeting pathways that converge on complementary signalling cascades is a strategy worthy of being exploited [33,34]. In agreement with this principle, our results demonstrate that simultaneous MEK and Mcl-1 inhibition induced by Zol, together with Doc-triggered apoptosis, could be key objectives in the treatment of hormone-sensitive prostate cancer.

The data reported in the present paper confirm those from other preclinical studies on the efficacy of Zol and Doc-Zol combinations [4,20,21] in the treatment of prostate cancer cells. They also highlight the importance of the schedule evaluated in hormone-sensitive cells using concentrations and exposures that could potentially occur in a clinical setting. Furthermore, considering the metastatic origin of the LNCaP cell line, our treatment option could also prove valuable in the management of hormone-sensitive metastatic cancer and in preventing the development of bone metastases regardless of hormone deprivation conditions, as suggested by Saad et al. [5].

## Conclusion

In conclusion, although further research is needed to widen our knowledge of the mechanisms underlying the cytotoxic synergistic interaction and to explore other drug schedules, the results from the present study suggest that, under androgen deprivation conditions, low Doc doses in concomitance with and followed by Zol could be a potentially useful first-line treatment for hormone-sensitive prostate cancer. This schedule would seem to be capable of reducing the tumor cell population and of rapidly eradicating hormone-resistant cells present in hormone-responsive tumors, without compromising the use of conventional-dose Doc.

## Abbreviations

Doc: Docetaxel; Zol: Zoledronic acid; SRB: Sulforhodamine B; OP: Optical density; RI: R index; LC: Lethal concentration.

## Competing interests

The authors declare that they have no competing interests.

## Authors' contributions

FF was responsible for study design, data analysis, and drafting the manuscript. WZ, DA and RS participated in the study design and acted as scientific advisors. FF, GB, SC, PU, IV and AT performed the *in vitro *experiments. All authors read and approved the final manuscript.
